# Impact of dietary supplementation of l-Arginine, l-Glutamine, and the combination of both on nursing performance of multiparous sows

**DOI:** 10.1093/tas/txac169

**Published:** 2022-12-25

**Authors:** Anna G Wessels, Aude Simongiovanni, Jürgen Zentek

**Affiliations:** Department of Veterinary Medicine, Institute of Animal Nutrition, Freie Universität Berlin, Berlin, Germany; METEX NOOVISTAGO, Paris, France; Department of Veterinary Medicine, Institute of Animal Nutrition, Freie Universität Berlin, Berlin, Germany

**Keywords:** arginine, glutamine, lactating sow, nursing performance, piglet, weaning homogeneity

## Abstract

Dietary supplementation with arginine (Arg) or glutamine (Gln) has been considered as an option to improve nursing performance in reproductive sows. This study investigated whether a low-level supplementation of Arg or Gln or a blend of both could modify milk nutrients and improve piglets’ growth beyond weaning. Seventy-two multiparous sows were assigned to four groups: one group fed a control diet, three treatment groups fed the control diet supplemented with either 0.35% Arg, 0.35% Gln, or both, from day 108 of gestation until weaning at day 26 of lactation. Immediately after birth, the litters were cross fostered to 13 piglets and monitored until 2 wk after weaning. Sows body condition and litter growth were assessed. Colostrum and milk samples were collected for nutrient analyses. Plasma concentrations of insulin-like growth factor 1 (IGF-1) around weaning were determined in sows and two representative piglets per litter. Supplementing Gln or the combination of Arg and Gln had no effect on the parameters studied. Arg supplementation increased weaning weight, while decreasing the variation of piglet weights 2 wk after weaning. There was no correlation with plasma IGF-1 since the hormone was not altered in sows or piglets. The colostral concentration of fat tended to increase in the Arg-group, whereas protein, lactose, energy, and polyamine concentrations remained unaffected. Milk samples obtained on day 12 and 25 of lactation were not influenced by dietary treatment. The data indicate that there might be a window of opportunity, explicitly at the onset of lactation, for dietary intervention by maternal dietary Arg supplementation.

## INTRODUCTION

Although arginine (Arg) and glutamine (Gln) are usually regarded as non-essential amino acids (AA), there is an increasing evidence that both should be considered as essential in several physiological and pathological states (Le Floc’h et al., 2018). For sows, early lactation represents a catabolic state due to the substantial increase of energy and nutrient requirements. Milk synthesis and many other metabolic processes, including mammary gland development require adequate AA and energy supply. Inadequate intake results in the mobilization of body protein with negative impact on the body constitution of sows (Clowes et al., 2005; Manso et al., 2012). Manso et al. (2012) reported that supplementing lactating sow diets with Gln or a mixture of Gln and glutamate (Glu) prevented the sows from constitutional losses and increased the milk concentrations of both AA. In general, the most abundant AA in mature milk of mammals is Gln and it is known to play important roles in the growth and development of the neonatal intestine (de Aquino et al., 2014). Studies with weaned piglets have also shown beneficial effects of supplemental Gln (López-Pedrosa et al., 2007; Wang et al., 2008; Duttlinger et al., 2020; Almeida et al., 2021). In addition, two studies have demonstrated improvements in growth and health of suckling piglets, either receiving twice daily additional Gln by gavage (Haynes et al., 2009) or receiving supplemental Gln and Glu in creep feed during the suckling period (Cabrera et al., 2013). Glutamate and Gln are also involved in Arg and proline (Pro) metabolism in a way that Arg is synthesized from citrulline that is derived from ornithine via catabolism of Pro or Glu (Wu, 1997). Next to Gln, sow milk is rich in Pro, which is used by neonate piglets to synthesize Arg. Nevertheless, in piglets and reproductive sows, Arg synthesis might be limiting for their optimal growth and reproduction, primarily due to the reduced expression of N-acetylglutamate synthase in enterocytes (Wu et al., 2004). This enzyme catalyzes the production of N-acetylglutamate (from Glu and acetyl-CoA) that is an allosteric activator of carbamoylphosphate synthase-I for the formation of citrulline and Arg (Wu and Morris, 1998). Young animals have a high requirement for Arg because of the utilization of Arg by multiple metabolic pathways (Mateo et al., 2008). However, Arg provision from sow’s milk might be insufficient for piglets’ protein deposition since estimates based on the supply of Arg from sow’s milk and the Arg requirement of piglets revealed that sow’s milk provides less than 40% of the daily requirement in 7-day-old suckling pigs (Wu et al., 2004; Le Floc’h et al., 2018). Both metabolic and growth data indicate that an Arg deficiency is a major factor limiting maximum weight gain of milk-fed piglets (Mateo et al., 2008). Therefore, increasing Arg intake in suckling piglets could be an effective strategy to promote their growth and immune system and prepare piglets for weaning, provided that maternal intake of extra dietary Arg resulted in accumulation of free Arg and Pro in milk. Next to the sows’ feed intake and the suckling intensity of piglets, milk production is further determined by the angiogenesis of mammary tissue and blood flow to the mammary glands, which enhance nutrient delivery to the mammary gland for milk synthesis (Trottier et al., 1997). Mammary blood flow and angiogenesis are regulated by Arg-derived nitric oxide (Kim and Wu, 2009). In addition, milk production is closely related to mammary gland growth, and Arg acts as a promoter for mammary gland growth (Pau and Milner, 1982). At a high dosage, Arg stimulates the secretion of prolactin, growth hormone, and polyamines, which are necessary for mammary development and nursing performance (Knopf et al., 1965; Davis, 1972). Based on this prior knowledge, we hypothesized that dietary supplementation of Arg, Gln, or a combination of both prevents constitutional losses of nursing sows and improves litter growth due to modulation of milk nutrients. Therefore, the current experiment focused on the reproductive performance of multiparous sows including milk nutrients, and growth performance of their litters beyond weaning as a function of maternal feeding.

## MATERIAL AND METHODS

### Animals and Treatments

All procedures involving handling and treatments of animals were approved by the local State Office of Health and Social Affairs (Landesamt für Gesundheit und Soziales, Berlin, Germany, LaGeSo Trial-ID: G 0281/18). The housing conditions of the animals complied with the applicable legal requirements, according to which the crates must have an area of 6.5 m^2^ and the weaned piglets up to a weight of 10 kg must be provided with an area of 0.15 m^2^.

**Figure 1.**
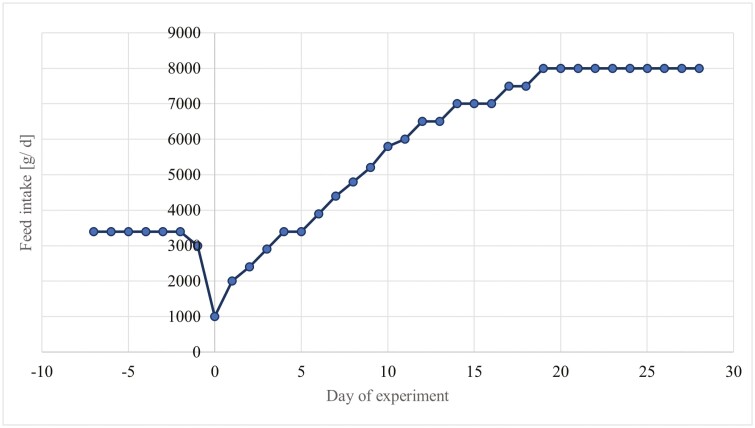
Feeding regime of the experimental sows.

Seven days (d) prior to their expected farrowing date, seventy-two gestating (DanBred × Piétrain) multiparous sows were evenly assigned to four treatment groups based on parity (4.9 ± 0.0) and back fat thickness (BFT; 16.3 ± 0.2 mm) and randomly placed in individual farrowing crates. The control group was fed a standard lactation diet. The treatment groups received the same diet as the control group supplemented on top with either 0.35 % Gln, 0.35 % Arg, or 0.35 % Arg + 0.35 % Gln from day 108 of gestation until weaning on day 26 post-partum (pp). The composition of the lactation diets is shown in Supplementary Table 1. The sows were fed twice daily at 0800 and 1600 h and had ad libitum access to drinking water. The total daily ration of the sows was adjusted from 3.5 kg/d prior to farrowing, to 1 kg at farrowing date, and incrementally to 8 kg/d from day 18 of lactation until weaning (Figure 1). Piglets [(DanBred × Piétrain) × Duroc] were delivered at term without artificial induction (day 114 ± 2 of gestation). All neonatal piglets were weighed within 8 h after birth and cross fostered within the treatment groups in order to standardize litters to 13 piglets with an average individual body weight (BW) of 1.4 ± 0.3 kg. Besides suckling sows’ milk, no additional creep feed was offered to the piglets during the nursing period. The piglets were weaned at 26 ± 2 day of age and allocated to flat deck pens in another barn of the same site. All litters per batch and maternal treatment were kept in the same pen. The piglets had ad libitum access to fresh drinking water and a commercial prestarter diet (Supplementary Table 2), which was offered in automatic feeders during the subsequent 14-d observation period.

On day 7 ante partum (ap) and on day 1, 12, and 26 pp, sows were weighed and BFT was assessed using a Lean-Meater (Renco Corporation, Golden Valley, MN, USA). The milk yield was calculated from the difference of litter weight at weaning and at cross-fostering multiplied with the factor 4.1 kg milk/kg weight gain according to GfE (2006). In order to evaluate the nursing performance of the sows, each piglet was weighed individually at birth, at day 7, 12, and 26 pp. In addition, the piglets were weighed at day 7 and 14 post-weaning.

In total, the experiment lasted 48 d for each sow including offspring. The experiment was conducted in six independent runs with none of the sows and their offspring used more than once.

### Sample Collection

On day 26 pp, blood samples were collected from the vena jugularis externa of 50% randomly chosen sows before the morning feeding (*n* = 9). One day prior to weaning two piglets per sow from the same sows that were sampled were used for blood sampling from the *vena cava cranialis*. Plasma was obtained by centrifugation (1,000 × *g* for 10 min at 4 °C) and was then stored at −80 °C.

The same sows used for blood sampling were milked manually within 12 h after initiation of farrowing (colostrum) and on day 12 and 25 pp (milk) without administration of oxytocin but with piglet removal to allow milk accumulation. Milk was collected from four functional glands on both sides of the mammary system. Each gland was milked into 50 mL Greiner Tubes until approximately 20 mL of milk was collected. The milking procedure lasted between 5 and 15 min depending on the individual. Samples from each sow and milking time point were pooled for chemical analysis and stored at −80 °C.

### Nitrogen and Amino Acid Quantification of the Diets

The nitrogen content was determined by DUMAS method (AFNOR Standard NF EN ISO 16634 – 1). The AA content of the diets was analyzed by a JLC-500/V AminoTac Amino Acid Analyzer (Jeol, Croissy-sur-Seine, France) in the laboratory of METEX NOOVISTAGO (Amiens, France) according to AFNOR (NF EN ISO 13903). To quantify methionine and cysteine, diet samples were oxidized with performic acid prior to hydrolyzation. Amino acids were separated by ion exchange chromatography and measured by photometric detection after derivatization with ninhydrin. Total tryptophan was analyzed by HPLC after an alkaline hydrolysis with barium hydroxide.

### Determination of Milk Nutrients and Related Metabolites

Crude fat and lactose concentration in milk were determined using standard procedures VDLUFA (VDLUFA 2010: VI 15.2.1, 20.2.3). Crude protein was analyzed using Dumas method (Dumas, 1831). Gross energy was calculated from milk nutrients multiplied with their respective factor: lactose, 16.4 kJ/g; fat, 38.9 kJ/g; protein, 23.8 kJ/g (Ramanau et al., 2004). Colostrum polyamines (i.e., putrescine, spermidine, and spermine) were analyzed by ion-exchange chromatography using a Biochrom 30 Amino Acid Analyzer (Biochrom Ltd., Cambridge, UK). Briefly, 1 mL colostrum or milk was incubated for 1 h at room temperature with an extraction buffer (20%, w/v trichloroacetic acid, 0.2% thiodipropionic acid, 0.5 mM hexamethylenediamine), then centrifuged (21,100 × *g* for 10 min at 4 °C) and filtered through 0.2-µm cellulose acetate membrane filter. Samples (25 μL injection volume) were separated on a 10-cm polyamine ion exchange column (Laborservice Onken, Gründau, Germany). Sodium citrate buffer (pH 7.2) was used as eluent and the amines were quantified after postcolumn ninhydrin derivatization by photometric detection at 570 nm.

### Determination of Plasma Concentrations of IGF-1

Sows and piglets’ plasma concentrations of insulin-like growth factor (IGF-1) were measured by quantitative enzyme-linked immunosorbent assay (ELISA) using a commercial kit (Ref: MD58011, IBL International GmbH, Hamburg, Germany) according to manufacturer’s protocol. All concentrations were determined in duplicate.

### Statistical Analyses

The statistical analyses were performed using the software package SPSS (IBM SPSS Version 25). Prior to one-way analysis of variance (ANOVA) based on treatment number, data were tested for normal distribution by Kolmogorov–Smirnov test (zootechnical performance) or Shapiro–Wilk test (physiological data) and variance homogeneity by Levene’s test. Covariates (parity number, sow, birth weight) were included to the statistical model in only case of significant impact on single target parameters. All treatment means were compared with each other using Tukey’s test (variance homogeneity) or Games-Howell test (variance heterogeneity). The statistical tests are shown in the footnotes of the respective data tables. Mean differences with a probability of *P* ≤ 0.05 were accepted as statistically significant and mean differences with 0.05 < *P* ≤ 0.10 were considered as trends.

## RESULTS

### Zootechnical Performance

The results on nursing performance of the sows are summarized in Table 1. The results on growth performance of the offspring after weaning are summarized in Table 2. The number of stillborn piglets was equal among the treatments. Litter size after cross-fostering (13.0 ± 0.0) and at weaning (11.7 ± 1.0) did not differ among the treatment groups. Dietary AA supplementation did not affect BW, feed intake, nursing losses, or days until return to estrus in the sows. However, the Arg-group tended to show the highest reduction of BFT during nursing (*P* < 0.10). At weaning, piglets from sows fed the Arg-supplemented diet had the highest BW among the experimental groups (*P* < 0.05). Accordingly, those piglets showed elevated BW gain during the second half of the nursing period. After weaning, the BW of piglets from sows fed the Arg-supplemented diet continued to be greater compared with piglets from control sows (*P* < 0.05) and had a lower coefficient of variation (*P* < 0.05). The BW gains did not differ from the control piglets after weaning (*P* > 0.05).

**Table 1.** Nursing performance of multiparous sows^1^

Treatment	Control	0.35% Gln	0.35% Arg	0.35% Arg + 0.35% Gln	SEM	*P*-value
**Farrowing parameters (*n*)**						
Stillborn	56	46	45	51	0.0	0.862
Mummies	3	3	3	4	0.0	0.978
Piglets born alive	286	281	293	280	0.0	0.823
Piglets after cross-fostering	234	234	234	234	0.0	–
Weaned piglets	210	212	209	209	0.0	0.960
Piglets born alive per litter	15.9	15.6	16.3	15.6	0.0	0.823
Initial piglets per litter	13.0	13.0	13.0	13.0	0.0	–
Weaned piglets per litter	11.7	11.8	11.6	11.8	0.12	0.953
Return to estrus rate	94 %	83 %	89 %	83 %	0.1	0.719
**Sows’ body weight, kg**						
d7^2^	292	316	300	289	6.2	0.313
d1	292	317	305	300	6.0	0.516
d12	289	306	295	293	5.1	0.716
d26	281	299	280	282	4.7	0.509
∆ d7 to d26	−0	−15	−21	−15	2.1	0.434
**Sows’ back fat thickness, mm**						
d7	16.00	16.39	16.29	16.56	0.213	0.831
d1	16.00	16.28	16.18	16.28	0.212	0.964
d12	15.89	16.06	16.00	16.11	0.201	0.984
d26	15.56	15.89	15.29	15.78	0.189	0.724
∆ d7 to d26	−0.44	−0.50	−1.00	−0.78	0.094	0.051
**Nursing parameters, kg**						
Litter weight (Farrowing)	21.2	21.7	21.0	21.2	0.52	0.959
Litter weight (Weaning)	88.2	89.3	92.9	86.8	1.47	0.526
Calculated milk yield	274	277	291	288	6.1	0.672
**Suckling period: Piglet body weight, kg**						
Birth weight_d0	1.35	1.39	1.35	1.36	0.013	0.453
BW_d7	2.5^ab^	2.6^b^	2.4^a^	2.5^ab^	0.01	0.007
BW_d12	3.9	3.9	3.9	3.8	0.01	0.383
Weaning weight_d26	7.6^a^	7.6^a^	8.1^b^	7.5^a^	0.11	0.004
**Suckling period: Piglet daily gain, g/d**						
d 1–7	168^b^	172^b^	152^a^	159^ab^	1.9	0.039
d 8–12	275^b^	262^ab^	279^b^	255^a^	3.2	0.041
d 12–26	242^a^	249^ab^	278^b^	244^a^	3.1	0.000
Average d 1–26	238^a^	244^ab^	253^b^	241^a^	2.0	0.001

Arg: arginine; Gln: glutamine; d7: seven days ante partum; d26: weaning; BW: average individual body weight.

^1^Data are presented as means (n_BW_ = 18 per treatment).

^2^Sows BW at 7 d ante partum was corrected by litter weight by subtracting the cumulated birthweight on the day of farrowing.

^a,b^ Values within a row with different superscripts differ significantly at *P* ≤ 0.05 (Tukey test).

**Table 2.** Growth performance of the offspring after weaning^1^

Treatment	Control	0.35% Gln	0.35% Arg	0.35% Arg + 0.35% Gln	SEM	*P*-value
**Individual body weight, kg**						
Weaning	7.6^a^	7.6^a^	8.1^b^	7.5^a^	0.11	0.004
d7	8.0^a^	7.9^a^	8.5^b^	8.0^a^	0.14	0.002
d14	9.4^a^	9.3^a^	9.9^b^	9.5^ab^	0.12	0.005
CV d14	0.1225^b^	0.1114^ab^	0.1089^a^	0.1217^b^	0.00170	0.006
**Individual daily gain, g/d**						
First week PW	55^ab^	49^a^	47^ab^	71^b^	1.8	0.002
Second week PW	201	188	194	208	3.4	0.087
Total	125^ab^	117^a^	125^ab^	136^b^	2.0	0.002
**Daily feed intake, g/d**						
First week PW	85	57	71	82	5.4	0.204
Second week PW	276	243	240	271	8	0.312
Total	181	150	155	176	6	0.184
**Gain-to-feed ratio, kg/kg**						
First week PW	0.65	0.78	0.55	0.86	0.051	0.070
Second week PW	0.74	0.77	0.83	0.69	0.008	0.207
Total	0.73	0.77	0.76	0.80	0.013	0.197

Arg: arginine; Gln: glutamine; d7 after weaning: d33 of the experiment; d14 after weaning: d40 of the experiment; CV: coefficient of variation; PW: post-weaning; SEM: standard error of the mean.

^1^Data are presented as means (Individual body weight and daily gain: *n* = 210; daily feed intake and Gain-to-feed ratio: *n* = 6).

^a,b^ Values within a row with different superscripts differ significantly at *P* ≤ 0.05 (Tukey test, Game–Howell test).

### Colostrum and Milk Parameters

Table 3 contains all data on Colostrum and milk parameters. No significant differences in milk nutrients were detected. Concentrations of fat, lactose, and protein were 8.4 ± 0.3 %, 5. 1 ± 0.1 %, and 5.2 ± 0.1 %, respectively for milk collected on day 12 and 8.4 ± 0.3 %, 4.6 ± 0.1 %, and 5.1 ± 0.1 %, respectively for milk collected on day 25. The gross energy was calculated to be 5.2 ± 0.1 MJ/kg in both milk of day 12 and 25 of lactation. The calculated milk yield did not differ among the treatments.

**Table 3.** Nutrient composition, gross energy, and polyamine concentrations of colostrum samples^1^

Treatment	Control	0.35% Gln	0.35% Arg	0.35% Arg + 0.35% Gln	SEM	*P*-value
Dry matter, %	24.95	23.92	21.09	21.23	0.67	0.445
Fat, %	5.47	5.81	7.07	3.87	0.423	0.053
Lactos, %	2.80	3.06	3.54	3.19	0.011	0.127
Protein, %	11.89	12.59	8.86	11.08	0.562	0.124
Gross energy, MJ/kg	5.2	5.6	5.5	4.7	0.18	0.272
Putrescine, µmol/L	48.5	64.7	58.6	56.9	3.83	0.588
Spermidine, µmol/L	12.6	9.3	9.2	10.4	0.68	0.330
Spermine, µmol/L	2.3	2.7	2.7	2.4	0.20	0.976

Arg: arginine; Gln: Glutamine; SEM: standard error of the mean.

^1^Data are presented as means (*n* = 9).

### Plasma Concentrations of IGF-1

Table 4 shows plasma concentrations of IGF-1 in piglets 1 d before weaning. Maternal supplementation with Arg, Gln, or both did not affect plasma concentrations of IGF-1 (*P* > 0.10). The concentrations were higher in piglets compared to sows (*P* < 0.05). In sows, IGF-1 was correlated to the calculated milk yield (*P* < 0.05, *r* = 0.415). In piglets, the IGF-1 concentration is correlated to the ancestry (i.e., mother sow; *P* < 0.05, *r* = 0.315), but not to maternal plasma IGF-1 concentration (*P* > 0.10).

**Table 4.** Plasma concentrations of Insulin-like growth factor 1 (IGF-1) in sows and piglets at weaning (ng/mL).

	Control	0.35% Gln	0.35% Arg	0.35% Arg + 0.35% Gln	SEM	*P*-value
Sows	164	118	166	149	12.3	0.513
Piglets	185	203	197	172	6.8	0.433

Arg: arginine; Gln: glutamine; SEM: standard error of the mean.

^1^Data are presented as means (*n* = 9).

## DISCUSSION

In the study presented here, relatively small supplementation levels were used. The study was designed in that way in order to avoid the high costs of a higher-percentage supplement. In this way, the price pressure to which agricultural producers are subject was considered. Conducting a study in a commercial setting has limitations that can simultaneously prove to be strengths: The experiment was conducted in six independent runs with none of the sows used more than once. After weaning, all piglets per batch and maternal treatment group were kept in one flat deck pen each. Regardless of the mothers’ treatment group, all piglet groups received the same commercial prestarter diet. Ideally, the piglets’ pen would be considered as the experimental unit because it is unknown whether the results on weaning weight were caused by the treatment of the pen. On the other hand, randomization was ensured by subdivision of the study into six batches. Therefore, the individual animal can be considered as the experimental unit even though piglets of a certain treatment were kept batch-wise in the same pen (Seo et al., 2018). According to Seo et al. (2018), a sufficient number of replicates is needed to obtain a reliable outcome from an experiment and to provide greater statistical power in order to detect a difference among treatments. This is true especially for commercial pig herds and experiments performed in various batches where the heterogeneity of animals is greater than in experimental animal husbandry. However, the cost of replicates is high in animal studies and in terms of the 3Rs principle, the smallest number of replicates is preferred as long as it is sufficient to detect a difference. For this reason, strong statements could be made in the present study regarding the final BW of the piglets, but not about their feed intake, which was documented pen-wise for practical reasons. In general, the observation period depicted in this field trial presents significant challenges for both the sows kept and their piglets. However, the systemic nutritional, social, or environmental stressors inherent in the commercial factory farming system were the same for all animals according to the *ceteris paribus* principle, so no influences on the efficacy of the supplements were expected.

The productivity of high prolific sows is basically restricted by their limited feed intake, which can lead to a catabolic state during lactation. Due to the practical nature of the present study, sows were fed according to the commercial farm’s general management and received 8 kg of their experimental diet daily at peak from day 18 of lactation. This aspect of the experimental design was based on the findings of previous studies in which the supplementation of 0.5% and 1% Arg HCl (Mateo et al., 2008; Cui et al., 2017) or 1% Gln (Yang et al., 2018) did not affect sows feed intake. However, Gao et al. (2020) showed recently that sows feed intake reached a doses-dependent plateau when Arg was fed in a ratio of 1.00:1.01 to dietary lysine. Therefore, consideration should be given on further dose–response studies to gain clarity on the impact of Arg and Gln supplementation on lactating sows feed intake. Sows body reserves act as a buffer of nutrients when the nutrients intake is insufficient (Vadmand et al., 2015). High rates of catabolism during lactation may lead to BW loss, decrease in BFT, metabolic disorders, or other diseases (Revell et al., 1998). In a recent review, Watford (2015) described that different doses of Gln and Gln with Glu supplementation attenuated some of the loss of lean body mass in the sow during lactation. And supplementation of Arg was shown to restore BW in lactating sows exposed to high ambient temperatures (Laspiur and Trottier, 2001) and to prevent BFT loss (Nuntapaitoon et al., 2018). This was not the case in the current study, where Gln supplementation had neither effect on BW nor on BFT while Arg-supplemented sows showed a trend for higher BFT loss with no effect on BW. Cui et al. (2017) reported similar results with numerically higher BFT and BW losses for sows supplemented with 0.5% Arg compared to 1% Arg or control. In some dose–response studies, no differences in sows BFT or BFT and BW in response to increasing dietary Arg supplementation from 0.0 to 0.4% or 0.0 to 0.79 % were detected (Gao et al., 2020; Hong et al., 2020).

Contrary to other studies that tested 1% Gln supplementation (Watford, 2015; Yang et al., 2018), we did not detect any effects of Gln supplementation on any of the parameters. Since dietary Gln is metabolized at approximately 70% of the intake by the enterocytes (Le Floc’h et al., 2018), it might be assumed that the dose used in the present experiment was not sufficient to exert significant effects in the sows’ periphery, as fat tissue or mammary gland nor was passed on to the piglets via milk. On the other hand, it could also be interpreted that the sows were already producing enough endogenous Gln to meet their needs, so that the additional Gln had no additional effect. Regarding the comparatively low BW loss during lactation, possibly also the catabolic state of the sows may not have been sufficient to see a Gln supplementation effect.

Kim et al. (2004) supplemented artificially reared suckling piglets directly with 0.2 and 0.4% Arg in their milk replacer and reported enhancements of daily gain and BW. Mateo et al. (2008) described a piglet growth improving effect even when sows were supplemented with 1% Arg during the entire lactation period. According to Mateo et al., the basic idea of the current experiment was to test the effects of Arg transition via milk at a dose closer to Kim et al. (2004) than to Mateo et al (2008) assuming that more piglets suckle at their mother than being reared artificially in practice. The current results on individual piglet BW at and after weaning confirm that this effect is valid with a lower level of supplementation. Based on the reduced coefficient of variation in the Arg group, this study also showed that low-dose Arg supplementation of sows has a positive effect on piglet uniformity even beyond weaning date. Litter weight gain is known to be correlated with milk production or nutrient concentrations (Noblet et al., 1987; Kirchgessner et al., 1991; King et al., 1993). Mateo et al. (2008) assumed that increased piglet or litter weight gain in sows extra-supplemented with 1% Arg may be indicative of increased milk production or nutrient concentrations. The calculated milk yields, which did not differ among the treatment groups in the current study, do not support this assumption. On the other hand, the colostrum of Arg-supplemented sows showed a trend for increased fat concentrations. The analyzed values reflect those reported by Hurley et al. (2015). Besides the elevated fat content, the Arg-supplemented sows showed also a strong trend for higher BFT losses. Therefore, it might be speculated that increased colostrum concentrations of fat originate from sow body fat reserves that were mobilized to a higher extent in the Arg-supplemented group. Indeed, Arg is involved in the lipid metabolism of pigs (Hu et al., 2015). As underlying mechanism Arg increases the expression of key proteins and enzymes (e.g., AMP-activated protein kinase [AMPK] and peroxisome proliferator-activated receptor-g coactivator 1a) responsible for mitochondrial biogenesis in brown adipose tissue, as well as substrate oxidation in insulin-sensitive tissues (e.g., mammary gland, skeletal muscle, liver, and white adipose tissue), thereby reducing white-fat mass (Wu et al., 2014). In addition, it was shown that dietary supplementation with 1% Arg increased intramuscular fat deposition by upregulating the mRNA levels of fatty acid synthase, thereby regulating fatty acid composition in the muscle (Tan et al., 2011). Milk fat and lactose are the main energy sources for piglet growth. For neonatal piglets, fat in colostrum delivers approximately 50% of the required energy (Tan et al., 2018). In contrast to the current data, another study revealed a trend for an elevated protein content and reduced lactose content of colostrum in sows supplemented with extra 25 g Arg per day, whereas colostrum fat was not affected (Krogh et al., 2016). The authors explained their observation by the reverse proportion of lactose to mammary secreta solids. Alternatively, they speculated that Arg might be a limiting factor for colostrum synthesis and explained the increased protein content in colostrum by the fulfillment of the elevated requirement for Arg at the onset of lactation (Krogh et al., 2016). Unfortunately, there is no information in the cited study on the actual Arg content of the control or treatment diets to allow comparison of control Arg levels with this study. Limited Arg content could also be due to the more restrictive feeding of sows around parturition in Krogh et al. (2016). Other possible explanations for the different changes in macronutrients in colostrum would be different housing conditions and associated immunological factors as well as the use of oxytocin in Krogh et al. (2016), which was omitted in our study. According to Quesnel and Farmer (2019), injection of oxytocin to sows in the early postpartum period delays the tightening of tight junctions in the mammary gland, prolonging the colostral phase and increasing concentrations of proteins such as IGF-I and IgG and IgA in early milk.

Arginine is incorporated into the mammary gland tissue to produce Pro, ornithine, urea, polyamines (spermine, spermidine, and putrescine), and nitric oxide (O’Quinn et al., 2002). Nitric oxide is a vasodilator and angiogenic factor that increases blood flow to the mammary gland tissue and can thus increase the supply of nutrients for milk production (Manjarin et al., 2014). Polyamines are synthesized from Arg via ornithine and are essential components with various biological functions including protein synthesis and regulation of lactogenesis. Their action is mainly via marked effects on the structure and function of genomic DNA molecules, and they have been shown to be essential for the growth and development of the neonatal small intestine (Manjarin et al., 2014). Based on this, an analysis of polyamine concentrations in colostrum was performed in the present study, which remained without significant results.

In suckling piglets, oral administration of polyamines or Pro has been shown to improve growth performance likely due to increased intestinal absorption, improved maturation of the intestinal mucosa and thus an improvement in epithelial restitution and barrier function after stress induction (Wang et al., 2015; Van Wettere et al., 2016). However, the results of colostrum analysis in our study did not show any effect of maternal supplementation with 0.35% of Arg on polyamine concentrations. Considering that 40% of ingested Arg is metabolized by the gut (Le Floc’h et al., 2018) and that polyamines synthesis from Arg in the mammary gland represents less than 1% (O’Quinn et al., 2002), it may explain the difficulty to measure the effect of low-dose Arg supplementation on colostrum polyamines concentration.

Furthermore, Arg stimulates secretion of important anabolic hormones such as insulin and growth hormone in pigs (Kim et al., 2004). It was demonstrated that dietary supplementation with Arg reduces the catabolic state of lactating sows by increasing their insulin status (Laspiur et al., 2006; Mateo et al., 2008). The effects of Arg on anabolic hormones such as insulin (Laspiur et al., 2006) may be responsible for an increase in the uptake of AA by the mammary gland (Laarveld et al., 1981). Besides insulin, Arg has also been shown to stimulate the release of growth hormone in growing pigs (Cochard et al., 1998). Growth hormone stimulates the release of IGF-1 in a linear manner (Etherton et al., 1987). Administration of growth hormone to gestating sows increased fetal weight on day 51 of gestation (Gatford et al., 2000) but had no effect on birth weight when administered the last 21 d of gestation (Kveragas et al., 1986). In the current study, piglet plasma concentrations of IGF-1 were measured at the end of the nursing period to determine if maternal supplementation with Arg would cause an increase in circulating IGF-1 levels that would translate to heavier piglets at weaning. However, there were no differences in circulating concentrations of plasma IGF-1 between the treatment groups at weaning. Possible reasons apart from insufficient transfer of Arg or IGF-1 to the piglets were discussed in a previous publication (Wessels et al., 2021). Likely valid in the current experiment is the mentioned down selection strategy for juvenile IGF-1 in modern pig breeding (Hermesch et al., 2001).

Results from the current study indicate that maternal supplementation of Arg during the lactation period improves piglet growth performance and homogeneity at and after weaning. At present, the practical strategies associated with Arg supplementation tend to follow the experimental data, where adding 1% Arg to lactation feed improves the performance of lactating sows and their litter (Mateo et al., 2008; Hong et al., 2020). Given the lower dose used in the current study and given the recent results of the dose–response study by Gao et al. (2020), a lower dose of supplementation may be used in practical supply of Arg to lactating sows.

In conclusion, the supplementation of Arg at 0.35% to lactating sows’ diet had a positive impact on piglet BW at weaning and 2 wk after weaning. During lactation, the piglets from Arg-supplemented sow group showed the highest BW gain, while sows mobilized more body fat. However, the tendency for the reduction in BFT had no effect on milk yield or return to estrus. The nursing performance in the Arg-supplemented sows might be related to a shift in colostrum nutrients, but not in milk nutrients. Most promising for the development of new feeding strategies is the effects of Arg supplementation in the lactating sow feed on piglet BW and homogeneity at weaning that are still visible two weeks after weaning. In the future, the use of Arg supplementation in lactating sow diets of different composition, as used in practical production, needs to be evaluated to fine-tune the adequate Arg dosage that will be cost-effective under on-farm conditions and with respect to litter size.

## Supplementary Material

txac169_suppl_Supplementary_Tables
