# Supplementary material for: Impact of dietary supplementation of l-Arginine, l-Glutamine, and the combination of both on nursing performance of multiparous sows
Source: Transl Anim Sci. 2022 Dec 25;7(1):txac169. doi: 10.1093/tas/txac169 (PMC9838099; doi:10.1093/tas/txac169)
Supplement: txac169_suppl_Supplementary_Tables [file txac169_suppl_supplementary_tables.docx]

Supplementary Table 1 Lactation diets fed to nursing sows from 7 d ante partum to weaning at d 26 (as-fed).

| **Ingredients (%)** | **Control** | **0.35 % Gln** | **0.35 % Arg** | **0.35 % Arg + 0.35 % Gln** |
| --- | --- | --- | --- | --- |
| Wheat | 35.10 | | | |
| Cereal brans | 13.70 | | | |
| Barley | 12.00 | | | |
| Barley grist | 10.00 | | | |
| Soy extraction meal | 6.00 | | | |
| Extruded cereals | 4.50 | | | |
| Triticale | 4.50 | | | |
| Rapeseed meal | 3.60 | | | |
| Rapeseed meal (heat-treated) | 3.00 | | | |
| Soy oil | 2.50 | | | |
| Calcium carbonate | 1.39 | | | |
| Sunflower seed extracted | 1.00 | | | |
| Sugar beet molasses | 0.50 | | | |
| Sodium chloride | 0.48 | | | |
| Monocalciumphosphate | 0.39 | | | |
| Vitamin-mineral premix^1^ | 1.34 | | | |
| Tested Amino Acid |  | 0.35 % Gln | 0.35 % Arg | 0.35 % Arg +  0.35 % Gln |
| **Calculated composition**^2^ | | | | |
| ME (MJ/ kg) | 13.2 | 13.2 | 13.3 | 13.3 |
| Crude protein (%) | 16.72 | 17.42 | 17.52 | 18.25 |
| **Total Arg (%)** | 0.89 | 0.89 | **1.24** | **1.24** |
| **Total Glx^3^ (%)** | 3.64 | **3.99** | 3.64 | **3.99** |
| Calcium (%) | 0.843 | 0.843 | 0.843 | 0.843 |
| Available phosphorus (%) | 0.467 | 0.467 | 0.467 | 0.467 |
| Total Lys (%) | 1.09 | | | |
| SID^4^ Lys (%) | 1.02 | | | |
| SID Thr:Lys | 0.59 | | | |
| SID Met:Lys | 0.23 | | | |
| SID Cys:Lys | 0.27 | | | |
| SID (Met+Cys):Lys | 0.51 | | | |
| SID Trp:Lys | 0.19 | | | |
| SID Ile:Lys | 0.52 | | | |
| SID Val:Lys | 0.66 | | | |
| SID Leu:Lys | 0.97 | | | |
| SID Arg:Lys | 0.79 | 0.79 | **1.10** | **1.10** |
| SID Glx:Lys | 3.26 | **3.58** | 3.26 | **3.58** |

^1^ Nutritional additives: 12000 I.E. Vit. A (3a672a/ Retinylacetate); 0.050 mg 25-Hydroxycholecalciferol (3a670a); 100 mg Vit. E (3a700/ all rac-alpha-Tocopherylacetate); 40 mg Vit. E Equiv. (from Polyphenols); 3.0 mg Vit. B (3a821) Thiamin mononitrate; 9.6 mg Vit. B2 (Riboflavin); 7.2 mg Vit. B6 (3a831/ Pyridoxine hydrochloride); 80 μg Vit. B12 (Cyanocobalamine); 4.8 mg Vit. K3 (3a711/ Menadion Nicotinamidbisulfite); 500 μg Biotin (3a880); 6.0 mg Folic acid (3a316); 400 mg Choline chloride (3a890); 40 mg Betaine (3a920/ Betaine anhydrate); 100 mg Fe (3b101/ Iron II carbonate); 30 mg Fe (3b106/ Fe II amino acid chelate-hydrate); 10 mg Cu (3b4.10/ Copper chelate of the hydroxy analogue of methionine); 0.35 mg Selen (E8/ Na-selenite); 0.05 mg Selen (3b815/ L-Selen methionine); 20 mg Mn (Manganese chelate of the hydroxy analogue of methionine); 6.00 mg Iodate (3b202/ Calcium iodate); 50 mg Zinc (3b610) Zinc chelate of the hydroxy analogue of methionine; 610 mg Ca salt of the hydroxy analogue of methionine (3c308).

Technological additives: citric acid (E330); 6 mg Sepiolit (E562); Antioxidants: 4.50 BHT (E321); 3.00 mg Propyl gallate (E310); Zootechnical additives: 500 FTU 6-Phytase (EC 3.1.3.26/ 4a24); Sensory additives: Aroma.

^2^ Calculated values based on INRAE feed tables (2018)

^3^ Glx represents the total amount of glutamic acid (Glu) and glutamine (Gln)

^4^ SID = standardized ileal digestible

Supplementary Table 2 *Commercial piglets prestarter diet fed from weaning until 14 d after weaning (as-fed).*

| **Ingredients:** Wheat, Corn (processed), Barley, Soy beans (thermically processed), Whey powder, Oatflakes, Soy protein concentrate, Wheat bran, Fish meal, Dextrose, Linseed expeller, Palm oil, Soy oil, Potato protein, Wheat bran (processed), Monocalcium phosphate, Hydrolysed pig protein, Coconut oil, Calcium carbonate, Sodium chloride, Yeast (extracted), Rapeseed (processed), C8, C10, C12 - fatty acids, additives^1^ | |
| --- | --- |
| **Declared composition** |  |
| ME (MJ/kg) | 14.50 |
| Crude protein (%) | 17.30 |
| Crude fat (%) | 7.40 |
| Crude fibre (%) | 3.00 |
| Crude ash (%) | 5.00 |
| Total Lys (%) | 1.45 |
| SID^2^ Lys | 1.36 |
| SID Thr:Lys | 0.62 |
| SID Met:Lys | 0.36 |
| SID Cys:Lys | 0.17 |
| SID (Met+Cys):Lys | 0.54 |
| SID Trp:Lys | 0.18 |
| SID Ile:Lys | 0.45 |
| SID Val:Lys | 0.52 |
| SID Leu:Lys | 0.85 |
| SID Arg:Lys | 0.65 |
| SID Glu:Lys | 2.26 |
| Calcium (%) | 0.66 |
| Phosphorus (%) | 0.56 |

^1^ Nutritional additives per kg: 12000 I.E. Vit. A (3a672a); 2000 I.E. Vit. D3 (E 671); 180 mg Vit. E as all rac-alpha-Tocopheryl Acetate (3a700); 150 mg Cu from Copper (II) sulfate pentahydrate (E 4); 10 mg Cu from Copper bilysinate (3b411); 100 mg Fe from Ferrous (II) sulfate monohydrate (E 1); 125 mg Fe from Ferrous (II) fumarate (E 1); 125 mg Fe from Ferrous chelate of glycine, hydrate (E 1); 100 mg Zn from zinc oxide (3b603); 20 mg Zn as Zinc chelate of glycine hydrate (3b607); 64 mg Mn from manganese oxide (E 5); 2 mg I from Calcium iodate anhydrous (3b202); 0.45 mg Se from Sodium selenite (E 8).

Antioxidants per kg: 21.0 mg Butylated hydroxytoluene (BHT/ E 321); 9.0 mg Propyl gallate (E 310); Zootechnical additives per kg: 1.0 x 10*9 KBE Enterococcus feacium DSM 7134 (4b1841/ Bonvital); 70 U Endo- 1,3(4)-Beta-Glucanase EC 3.2.1.6; 80 U Endo- 1,4-Beta-Glucanase EC 3.2.1.4; 270.0 I.U. Endo- 1,4-β Xylanase EC 3.2.1.8; 0.50% Benzoic acid (4d210); Sensory additives: 1.5 mg Neohesperidin dihydrochalcone (E 959); 130 mg Sodium saccharin (E 954iii).

^2^ SID = standardized ileal digestible
